# Comparison of Apical Sealing Capacity of ActiV GP/Glass Ionomer Sealer Versus Resilon/RealSeal and Gutta Percha/AH plus Sealers

**DOI:** 10.7759/cureus.49931

**Published:** 2023-12-04

**Authors:** Unmesh Khanvilkar, Jyothi Dundappa, Nitu Chaubey, Anju Jha, Ajay Paliwal, Rahul Kumar

**Affiliations:** 1 Department of Conservative Dentistry and Endodontics, Yogita Dental College and Hospital, Khed, IND; 2 Department of Periodontology, Kalinga Institute of Dental Sciences, Bhubaneswar, IND; 3 Department of Conservative Dentistry and Endodontics, Purvanchal Institute of Dental Sciences, Gorakhpur, IND; 4 Department of Pediatric and Preventive Dentistry, Patna Dental College and Hospital, Patna, IND; 5 Department of Conservative Dentistry and Endodontics, Saraswati Dental College, Lucknow, IND; 6 Department of Orthodontics, Sanjeevani Dental Clinic, Patna, IND

**Keywords:** endodontics, gutta percha/ah plus, resilon/realseal, apical sealing capacity, activ gp/gisealer

## Abstract

Background: In recent years, concerns have arisen regarding the sealing efficacy of traditional root canal obturation materials such as gutta-percha (GP) and various sealers. The resin-based obturation complexes like RealSeal^TM^ (SybronEndo, Orange, CA) and Resilon^TM^ (Resilon Research LLC, Madison, CT) had been developed as replacements for conventional obturation materials, gutta-percha, and various types of sealers. Additionally, ActiV GP^TM^ (Brasseler USA, Savannah, GA), a glass-ionomer-based obturation system, has gained attention.

Aim: This in vitro study's objective was to evaluate ActiV GP^TM^/GI (glass ionomer) sealer's sealing capacity and compare it to that of Resilon^TM^/RealSeal ^TM^ as well as gutta-percha/AH Plus^TM^ (Dentsply International Inc, York, PA) taken as a control.

Methods and materials: In this in vitro investigation, 90 freshly excised single-rooted human premolars of the mandible were chosen. According to the substance used to seal the root canals, the teeth were randomly separated into three separate groups (n=30). Group 1: The ActiV GP^TM^ obturation system was used to seal and obturate the canals. Group 2: Resilon^TM^/RealSeal^TM ^was used to obturate and block the canals. Group 3: GP points and AH Plus^TM^ were used to seal the canals employing the lateral condensation methodology. All of the root surfaces-aside from the last 2 mm of the root-were painted with two coats of nail polish before being submerged in 2% methylene blue for a duration of 24 hours at a temperature of 37°C. The highest amount of dye leakage was determined using a stereomicroscope at 20X magnification.

Results: The extent of dye penetration at the apical region in specimens of the ActiV GP^TM^ category was 4.93±1.48 mm. The depth of dye penetration at the apical region in specimens of the Resilon^TM^ category was 2.78 ±1.62 mm. The extension of penetration of dye was 0.48± 0.46 mm in specimens of the GP/AH Plus^TM^ category. The degree of microlikeage was maximum in ActiV GP^TM^ specimens and it was minimum in GP/AH Plus^TM^ specimens. The microlikeage in specimens of the Resilon category was greater than GP/AH plus^TM^ while it was lower than ActiV GP^TM^ specimens. The observations were statistically meaningful (p<0.001).

Conclusion: Based on the results of the dye penetration examination, it can be concluded that the root canal wall sealing capabilities of ActiV GP^TM^ with GI Sealer were inferior to those of GP/AH Plus^TM^ and Resilon^TM^ with RealSeal^TM^.

## Introduction

Endodontic therapy aims to achieve thorough cleaning, shaping, and complete filling of the root canal system in all three dimensions [1,2], with the primary objective of creating a hermetic seal to prevent the ingress of microorganisms and their byproducts into the periapical tissues [3]. Inadequate obturation of the root canal space significantly contributes to endodontic treatment failure, accounting for approximately 60% of such cases [3]. Various endodontic materials have been employed to obturate the root canal, typically involving a core material and a sealer [3,4]. Due to its qualities as a biocompatible, chemically inactive, dimensionally stable, and pliable material, gutta-percha has been utilized and is still widely regarded as the most preferred material [4,5].

Concerns over the inadequate sealing abilities of gutta-percha, various sealers, and standard root-filling solutions have grown in recent years [6,7]. One of the most recent advancements in endodontics is the development of obturating materials that can adhere to dentin at the wall of the root canal to close interfacial voids coronally and apically. Hybrid layer creation has been used to apply dentin bonding properties to obturating substances, which were adapted via restoration dentistry [8]. The production of glass ionomer (GI) based root canal sealers has also embraced the use of glass ionomer cement (GICs), which chemically links to dentin by a process of ion exchange and the development of a transition layer across the dentin material contact [9]. 

The manufacturers of root canal obturation substances created an uninterrupted series of attached interfaces using adhesive methods between dentin and sealer extending up to the central obturation substance, as well as throughout the coronal portion towards the apical third of the canal domain, including all of the discrepancies of root canal like isthmuses, cul de sacs, and fins. Monoblock is formed in the canal of the root as a consequence of this conceptual idea [10], which if effective, should totally eradicate interfacial discrepancies, generate a flawless coronal as well as apical closure, and avoid another infection of the space within the canal following the endodontic therapy [11]. As substitutes for traditional obturation materials, gutta-percha and various types of sealers, resin-based obturation complexes like RealSeal^TM^ (SybronEndo, Orange, CA) and Resilon^TM^ (Resilon Research LLC, Madison, CT) have been developed [12].

Resilon^TM^ is said to be the first obturation system with the capacity to create a "monoblock" between the obturation material and the canal walls [13]. Resilon^TM^ belongs to polycaprolactone polymer material made up of bioactive glass containing radiopaque fillers. On one aspect, the resin sealer clings to a Resilon core, while on the other, it bonds to the surface of the etched root dentine [12]. A new GI-based obturation system is called ActiV GP^TM ^(Brasseler USA, Savannah, GA). According to the producer, the product has superior time to operate, radiopacity, and operating characteristics over earlier models [14]. ActiV GP^TM^ is made up of gutta-percha structures with GI coated on the outside. The aforementioned cones come in 0.04 and 0.06 tapering cone diameters, and their exact laser-defined dimensions guarantee a better fit. These cones are employed in conjunction with a GI sealer, forming a single cone to create an attachment between the master cone and the dentin at the walls of the root canal [12]. This in vitro study's objective was to evaluate ActiV GP/GI sealer's sealing capacity and compare it to that of Resilon^TM^/RealSeal^TM^ as well as gutta-percha/AH Plus (Dentsply International Inc, York, PA) taken as a reference.

## Materials and methods

In this in vitro investigation, 90 freshly extracted single-rooted human premolars of the mandible were chosen. According to a visual inspection of the mesiodistal and buccolingual radiographs, the teeth were morphologically compared. Each tooth chosen for the present research had one canal, a partially formed root that wasn't fully dilacerated and was devoid of resorption, cavities, fractures, and restorations. According to the substance used to seal the root canals, the teeth were randomly separated into three separate groups (n=30). Scaling and polishing were used to get rid of any calculus, bone fragments, or attached soft tissues. To get access to the root canal, the crowns of teeth were cut in half at the cementoenamel junction with a fast-speed diamond cutting bur (Dentsply Sirona, USA). Ethical clearance was obtained from Yogita Dental College and Hospital Institutional Review Board (IRB) with approval number IEC/YDCH/2019/222.

The optimal length for each root canal was objectively determined by inserting a size 10 K endodontic file (Dentsply Sirona, USA) until the file tip was visible at the apical foramen. The operational length was set at 1 mm less than the apex. Employing a crown-down approach and K3 NiTi rotary files (Dentsply Sirona, USA), the canal networks were biomechanically prepared till the length of operation until a size 40 having taper 06 was achieved. After using every device, one milliliter of sodium hydrochloride (5.25%) was administered as an endodontic irrigant, followed by three milliliters of 17% EDTA (Dentsply Sirona, USA) for one minute to eradicate the smear layer. Five milliliters of distilled water were used for the last rinse. After that, sterile paper points (Dentsply Sirona, USA) were used to dry the root canals. Every prepared root canal was sealed off as follows:

Group 1: The ActiV GP^TM^ obturation apparatus (Densply Sirona, USA) was used to seal and obturate the canals. In the ActiV GP category, a single cone method was employed. ActiV GP sealer was applied to the master cone (40/06), which was then slowly inserted into the root canal once the desired working length was attained. The master cone remained 2 mm below the canal orifice after setup.

Group 2: Resilon^TM^/RealSeal^TM ^was used to obturate and block the canals. Obturation was carried out in accordance with the manufacturer's procedure instructions in the Resilon^TM^ category. Employing sterile paper points, the self-etching primer (RealSeal Primer) was inserted into the root canal, with sterile paper points to coat the root canal walls, and excess primer was removed with sterile paper points.

The single-cone approach was used to fill the canals. RealSeal sealer was applied to the main cone (40/06), which was then slowly inserted into the root canals until it approached its operational length. After the obturation was finished, the coronal surface underwent a 40-second light cure. The primary cone was 2 mm below the canal orifice once it had been set.

Group 3: GP points and AH Plus^TM ^were used to plug the canals employing the lateral condensation methodology. A standardised size 40 GP master point, was chosen, inserted into the root canal to its full working length, and examined for tugback requirements. AH Plus^TM^ sealer was applied to the wall of the canal employing a K file size #25 in an anti-clockwise rotation after being mixed in accordance with the manufacturer's instructions. Once the working length was attained, the master point was progressively inserted into the root canal after being coated with a sealer. We carried out lateral condensation with standardized finger spreaders. The GP cones were 2 mm below the canal aperture after the obturation was finished. This technique relies on the sealer being applied both to the canal walls and the master cone to create a seal that minimizes the risk of leakage and bacterial penetration.

Following root canal obturation, GIC (Dentsply Sirona, USA) was used to seal the access cavities, which were then kept at 37°C temperature and 100% humidity for seven days. After that, all of the root surfaces-aside from the last 2 mm of the root-were painted with two coats of nail polish before being submerged in 2 percent methylene blue for 24 hours at a temperature of 37°C. The highest amount of dye leakage was determined using a stereomicroscope at 20X magnification (OPTIKA STX, USA) after each root was divided and separated longitudinally in a buccolingual orientation with a diamond disc (DFS Diamon, USA) of 0.3 mm thickness employing a slow speed handpiece using water cooling. Escobar et al.'s [15] criteria were applied to analyze the infiltration proportions in the present in vitro investigation to measure the apical leakage.

This research was completed utilizing SPSS software, version 20.0 (IBM Corp., Armonk, NY). The data were analyzed with descriptive statistical techniques utilizing the Kruskal-Wallis and Mann-Whitney analyses (p = 0.05). The significance threshold was set at 0.05.

## Results

The maximum proportion of specimens (83.3%) showed a score of 2 for penetration of dye at the apical region, while 16.7% of specimens showed a score of 1 in the group of ActiV GP^TM^ according to the assessment carried out by Escobar et al. parameters. The maximum proportion of specimens (36.63%) showed a score of 1 for penetration of dye at the apical region, while 30% of specimens showed a score of 0 and 33.33% of specimens showed a score of 2 in the group of Resilon. All specimens (100%) in the GP/AH Plus^TM^ group had a score of 2 (Table 1).

**Table 1:** Scores for penetration of dye at apical region assessed according to Escobar et al.’s criteria N: Number of specimens; %: Percentage. Escobar et al. [15]

Scores	Group 1: ActiV GP^TM^ 'N (%)'	Group 2: Resilon^TM^ 'N (%)'	Group 3: GP/AH Plus ^TM^ 'N (%)'
0	0	9 (30)	0
1	5 (16.7)	11(36.63)	0
2	25 ( 83.3)	10 (33.33)	30 (100)
Total	30	30	30

These percentages represent the outcomes of the assessment of dye penetration in each group, providing insight into the effectiveness of the different obturation materials or techniques in preventing dye penetration at the apical region of the root canals.

The extent of dye penetration at the apical region in the ActiV GP^TM^ group specimens was 4.93±1.48 mm. The depth of dye penetration at the apical region in specimens of the Resilon group was 2.78 ±1.62 mm. The extension of penetration of dye was 0.48± 0.46 mm in specimens of the GP/AH Plus^TM^ group. The degree of microleakage was maximum in ActiV GP^TM^ specimens and minimum in GP/AH Plus^TM^ specimens. The microlikeage in specimens of the Resilon group was greater than GP/AH Plus^TM^ and lower than ActiV GP^TM^ specimens. The observations were statistically significant (p<0.001) (Table 2).

**Table 2:** Extent of penetration of dye at apical region (mm) A p-value was considered significant at p<0.05; SD: standard deviation in millimeters; Mean value in millimeters.

Variables	Group 1: ActiV GP^TM^	Group 2: Resilon^TM^	Group 3: GP/AH Plus^TM^
Minimum (mm)	2.01	0.5	0.1
Maximum (mm)	6.92	5.91	0.98
Mean (mm)±SD	4.93±1.48	2.78±1.62	0.48±0.46
p-value	0.001		

## Discussion

Endodontics has introduced a variety of obturation materials, some of which are adaptations from restorative dentistry, while others are innovative materials with novel approaches [16]. Since it is known that GP does not give the optimal bonding to dentin of the root canal, numerous investigations are being done to develop substitute materials for forming an adequate apical seal and physically supporting the root structure [17-19]. Although GIC-based sealers can firmly cling to dentinal walls, they are unable to bind to GP points. As a result, after thorough setting up, a gap that separates the sealer as well as gutta-percha persists through which fluids and germs can pass [13].

ActiV GP^TM^ is a glass-ionomer-based obturation system, claimed by the manufacturer to surpass previous models in terms of working time, radiopacity, and handling characteristics [14]. Gutta-percha structures with GI coating on the outside make up ActiV GP^TM^. The aforementioned cones are available in 0.04 and 0.06 tapering cone diameters, and a better fit is ensured by their exact laser-defined dimensions. To establish a connection between the master cone and the dentin at the root canal walls, these cones are used in conjunction with a GI sealer to produce a single cone [12]. This in vitro study's objective was to evaluate ActiV GP^TM^/GI sealer's sealing capacity and compare it to that of Resilon^TM^/RealSeal^TM^ as well as gutta-percha/AH Plus^TM^ taken as a reference.

The results of our investigation were in opposition to those of Kassar et al. [14], who showed no difference in leakage among teeth sealed with ActiV GP^TM^/GI sealer compared to GP/AH Plus^TM^. After a seven-day gap, Garg TG's study demonstrated much less leakage in the ActiV GP^TM^ system category compared to the gutta-percha/AH Plus^TM^ category and the Resilon^TM^/Epiphany category. At the conclusion of the fourth week, there was not a statistically significant distinction in the amount of leakage between each of the three tested substances: Resilon^TM^/Epiphany, ActiV GP^TM^ system, and gutta-percha/AH Plus^TM^ [20]. In the present study, a highly significant difference between the GP/AH Plus^TM^ group and the ActiV GP^TM^ group was observed due to ActiV GP^TM^/GI sealer's shrinkage during setting and the GI sealer's nonhomogeneous mix, which is doubtful and could negatively affect its properties.

In recent years, concerns have grown regarding the inadequate sealing properties of traditional materials such as gutta-percha, various sealers, and standard root-filling techniques [16,17]. Through the construction of hybrid layers, obturating materials that were modified by restoration dentistry have been given dentin bonding characteristics [18]. Glass ionomer cements (GICs), which chemically bind to dentin through a process of ion exchange and the establishment of a transition layer across the dentin material contact, have also been welcomed by the production of GI based root canal sealers [19]. 

Gutta-percha has been used and is still recognized as the most favored material due to its properties as a biocompatible, chemically inactive, dimensionally stable, and malleable material [21-25]. Adhesive systems have demonstrated trustworthy outcomes in restorative dentistry, including long-term execution and reduced procedure sensitivity. The debate over the effectiveness of adhesive systems within the root canal in endodontics continues due to the drawbacks of adhering to radicular dentine [26]. It has been demonstrated that a number of circumstances have a negative impact on resin-based obturation materials adherence. To ensure optimal bonding and sealing with resin-based obturation materials, it's important for clinicians to follow proper canal preparation, cleaning, and disinfection protocols while considering factors that may affect material adherence. Another drawback of dentin binding is the resin's tendency to deteriorate over time as a result of functional stresses or partial resin entry into demineralized dentin, which allows fluid to pass across the hybrid zone and undamaged dentin [27,28]. The distinction between the GP/AH Plus^TM^ group and the ActiV GP^TM^ group was found to be highly significant. This outcome may be attributable to ActiV GP^TM^/GI sealer shrinkage during setting as well as a dubious nonhomogeneous GI sealer mixture that may have adverse effects on the sealer's characteristics. A thick coating of sealer may be present at the interface area between the dentine surface and the filling material thanks to the ActiV GP^TM^/GI sealer system's single-cone approach, in which the master cone closely resembles the configuration of NiTi rotary instruments [14]. A wider thicker covering of sealer could result in gaps due to shrinking during the setting reaction, which has been shown to have a detrimental impact on sealing ability [22].

Resin-based obturation complexes like RealSeal^TM^ and Resilon^TM^ have been developed as replacements for conventional obturation materials, gutta-percha, and various types of sealers [24,25]. Resilon^TM^, the first obturation device to have this capability, is believed to establish a "monoblock" between the obturation substance and the wall of the root canal [20,21]. Resilon^TM^ is a radiopaque filler-filled bioactive glass-based polycaprolactone polymer material. The resin sealer adheres to a Resilon^TM^ core on one side and the surface of the etched root dentine on the other [14,15].

Numerous assessments on various elements of this material have been released since its launch in 2004. Because there was a statistically noteworthy distinction between the Resilon^TM^ category and the other categories, the results of the current study did not agree with those of earlier research [14,20,21]. However, according to other studies, various obturation systems, such as Guttaflow and Resilon^TM^ cones with Epiphany, offer a better barrier against microbiological infiltration compared to gutta-percha/AH plus^TM^ sealer [24,25].

One limitation of this study is that it was conducted in vitro using extracted human premolars, which may not fully replicate the complex and dynamic conditions of the human oral environment. In vivo factors such as pulpal pressure, tissue response, and the presence of bacteria were not taken into account, potentially influencing the sealing properties differently than observed in this controlled laboratory setting. Additionally, the use of methylene blue dye penetration as the sole method for assessing sealing capacity may not entirely represent clinical outcomes, as other factors like microbial leakage and long-term stability were not considered. Scanning electron microscopy (SEM) can be used to examine the surface morphology and integrity of obturation materials and the interface between the material and the canal walls at a microscopic level. Confocal laser scanning microscopy is an advanced imaging technique that allows for three-dimensional visualization of the obturated root canal system.

## Conclusions

In summary, the root canal sealing performance of ActiV GP^TM^/GI sealer was found to be less effective when compared to GP/AH Plus^TM^ and Resilon^TM^/Real Seal^TM^. Furthermore, the Resilon^TM^/Real Seal^TM^ obturation system, designed as an alternative to gutta-percha for root filling, did not exhibit superior sealing capabilities compared to the conventional combination of gutta-percha cones and AH Plus^TM^ sealer. Interestingly, the lateral condensation obturation technique demonstrated improved sealing properties when contrasted with the single cone technique. It is noteworthy to emphasize that, at present, there is no single root canal obturation material that comprehensively fulfills all the criteria for achieving a flawless root canal seal in endodontic practice.
